# Intraoperative real-time three-dimensional transesophageal echocardiography as a precise navigator for a successful complicated postoperative left ventricular pseudoaneurysm repair: a case report

**DOI:** 10.1186/s40981-019-0261-y

**Published:** 2019-06-21

**Authors:** Futaba Miyoshi, Yusuke Seino, Minoru Nomura, Makoto Ozaki

**Affiliations:** Department of Anesthesiology, Tokyo Women’s Medical University, 8-1 Kawada-cho, Shinjuku-ku, Tokyo, Japan

**Keywords:** Left ventricular pseudoaneurysm, Aortic root replacement, Real-time three-dimensional transesophageal echocardiography

## Abstract

**Background:**

Left ventricular pseudoaneurysm (LV-PAN) formation is a rare complication after cardiac surgery and mainly occurs after mitral valve surgery. Echocardiography plays a critical role in the assessment of rupture location, orifice geometry, and anatomical relationship with surrounding structures.

**Case presentation:**

A 56-year-old man presented with LV-PAN formation 1 year after aortic root replacement combined with aortic replacement despite the lack of direct manipulation of the rupture site in the procedure and postoperative myocardial infarction. Intraoperative real-time three-dimensional transesophageal echocardiography (RT 3-D TEE) during surgical repair of the LV-PAN facilitated understanding of the shape of the LV-PAN orifice and the exact anatomical relationship between the rupture site and the posteromedial papillary muscle. Information sharing with surgeons contributed to avoiding direct papillary muscle injury and thus mitral valve deformation.

**Conclusion:**

LV-PAN formation after cardiac surgery can present without direct manipulation of the rupture site and major coronary lesion. Intraoperative RT 3-D TEE can facilitate better understanding of the anatomical relationship between the rupture site and the posteromedial papillary muscle and allow for information sharing to avoid complications during surgical repair.

## Background

Left ventricular (LV) pseudoaneurysm (PAN) formation is a rare complication of cardiac operations and mainly occurs as a result of LV free wall rupture after mitral valve surgery [1, 2]. The common causes of LV-PAN formation are transmural myocardial infarction, LV trauma, infection, and prior cardiac surgery, mainly mitral valve replacement; 33–58% of the LV-PAN cases results from cardiac surgery [1–4]. The PAN location depends on the operative procedure; for example, the PAN is formed in the posterior subannular region of the mitral valve after mitral surgery [2]. The treatment of LV-PAN consists of surgical repair; however, the surgical mortality is high, ranging from 7–20% [1, 2, 5]. Echocardiography plays a critical role in the assessment of rupture location, orifice geometry, and anatomical relationship with surrounding structures. Although the superiority of real-time three-dimensional (3D) echocardiography over two-dimensional (2D) echocardiography in the assessment of LV-PAN has been reported, the efficient application of intraoperative real-time 3D transesophageal echocardiography (RT 3D TEE) in the surgical repair of LV-PAN is still unestablished [6, 7]. We present a case of LV pseudoaneurysm (LV-PAN) formation without a major coronary lesion that developed 1 year after aortic root replacement combined with ascending aorta and hemiarch replacement, regardless of no findings of PAN and myocardial ischemia in the perioperative period. Intraoperative real-time three-dimensional transesophageal echocardiography (RT 3-D TEE) during the surgical repair of LV-PAN could contribute to avoiding surgical complications and better understanding of the anatomical structure and information sharing with cardiac surgeons.

## Case presentation

A 56-year-old man presented with shoulder pain at rest and ST-segment elevation in leads II, III, aVF, V5, and V6. He had a history of valve-sparing aortic root replacement (the David procedure) combined with ascending aorta and hemiarch replacement for annuloaortic ectasia 1 year prior to presentation. His LV function immediately after surgery was normal without any regional wall motion abnormality. Coronary angiography was performed under the suspicion of acute coronary syndrome; 90% stenosis of the diagonal branch of the left anterior descending artery was detected. Transthoracic echocardiography (TTE) revealed a 17-mm perforation in the inferior wall of the LV and a PAN with a diameter of 7 cm. The LV dimensions and wall motion were normal. Mild mitral regurgitation was detected. Similar findings were obtained using left ventriculography and cardiac computed tomography.

Because the patient was hemodynamically stable, with a low dose catecholamine administered soon after admission to the intensive care unit, elective surgical repair was planned due to the risk of redo surgery. However, his LV function and respiratory status rapidly deteriorated. He was intubated and placed on mechanical ventilation on hospital day 3. Intraaortic balloon pumping (IABP) was initiated due to low cardiac output syndrome on hospital day 5, and continuous hemodiafiltration was started on hospital day 7 due to acute kidney injury. As the orifice and size of the LV-PAN were enlarged, urgent surgical repair was performed on hospital day 11.

**Fig. 1:**
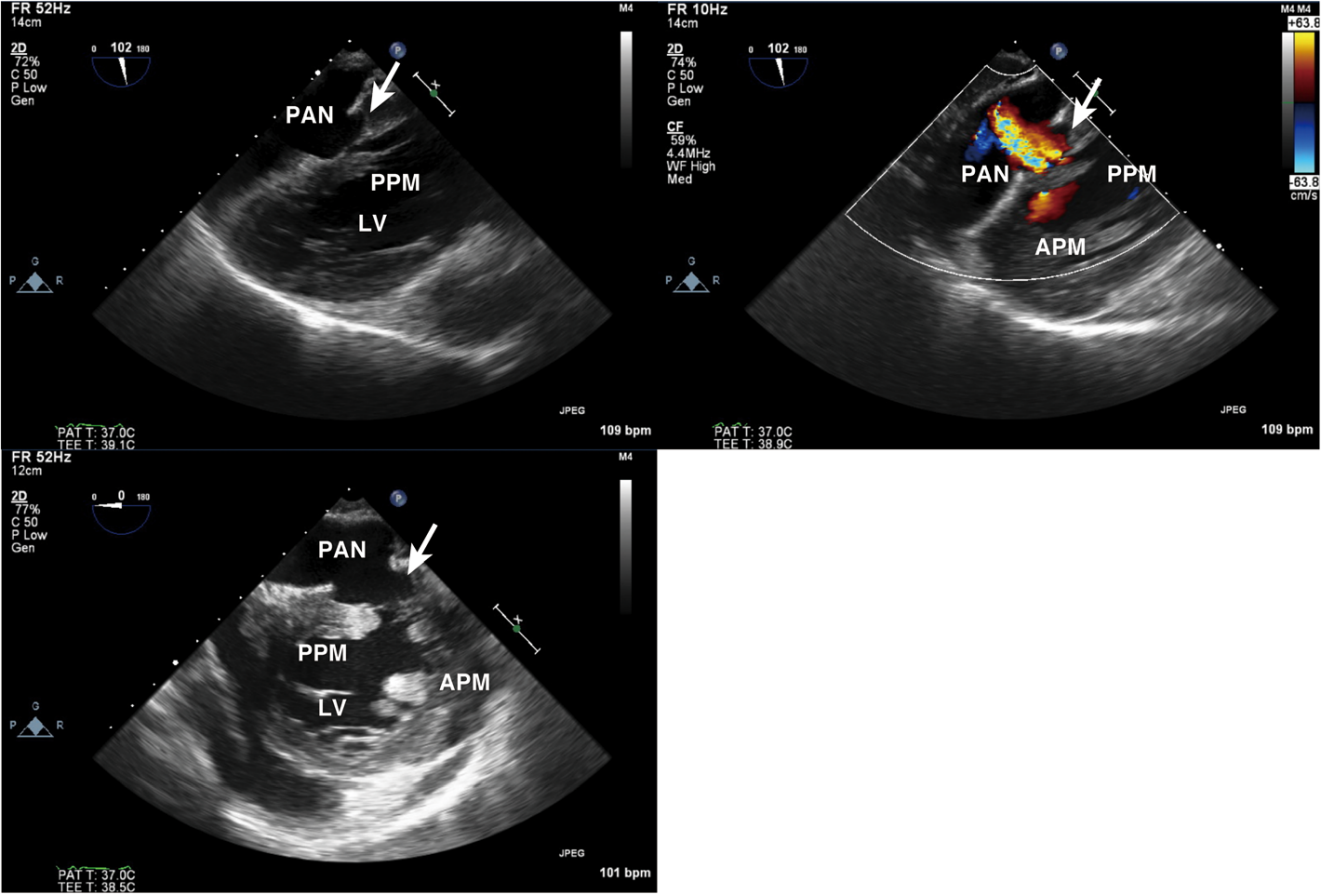
Transgastric long-axis view (two-dimensional, upper left; color flow Doppler, upper right) and mid-papillary short-axis view (lower). The orifice of the pseudoaneurysm is located in the inferior wall of the left ventricle (arrow). APM anterolateral papillary muscle, LV left ventricle, PAN pseudoaneurysm, PPM posteromedial papillary muscle

**Fig. 2:**
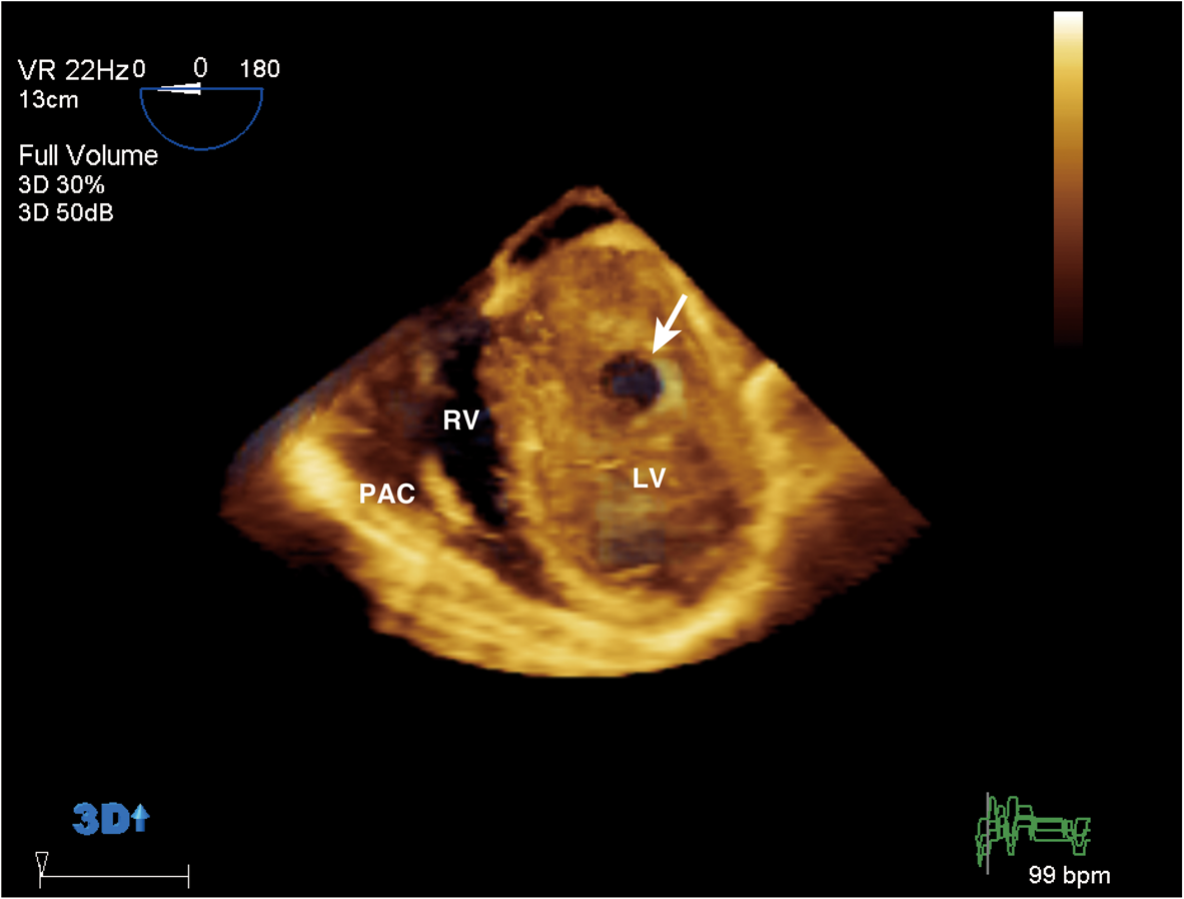
Three-dimensional view of the orifice of the pseudoaneurysm from inside the LV (arrow). The orifice of the pseudoaneurysm was located in the inferior wall. This three-dimensional view clearly shows the shape of the orifice. LV left ventricle, PAC pulmonary artery catheter, RV right ventricle

**Fig. 3:**
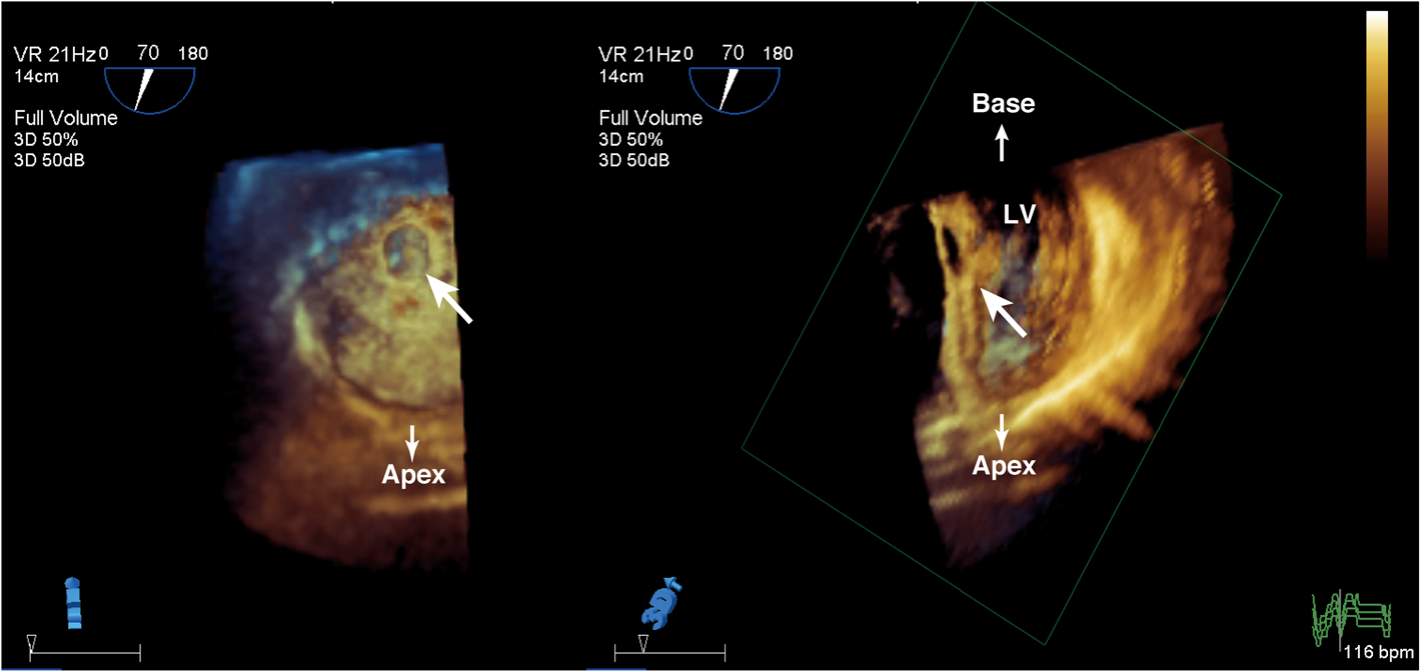
Three-dimensional images of the orifice of the pseudoaneurysm from outside (left) and inside (right). The images were obtained in the transgastric view. The attachment site of the posteromedial papillary muscle (arrows) is located very close to the orifice. This finding suggests that repair of the left ventricular pseudoaneurysm might influence papillary muscle function

General anesthesia was induced and maintained using sevoflurane, propofol, remifentanil, and rocuronium. Hemodynamics was maintained with dopamine at 2 μg/kg/min, dobutamine at 4 μg/kg/min, and milrinone at 0.35 μg/kg/min, all of which had been administered before surgery. Intraoperative two-dimensional (2-D) TEE revealed LV free wall rupture with a large echo-free space in the inferior wall (Fig. 1). The RT 3-D TEE image clearly showed that the orifice of the PAN was located at the mid-inferior segment of the LV (Fig. 2), adjacent to the attachment site of the posteromedial papillary muscle (Fig. 3). The orifice of the PAN was located at the basal side of the insertion of the posteromedial papillary muscle; thus, we informed the cardiac surgeons that the repair procedure might affect the function of the posteromedial papillary muscle. Both side orifice images from PAN cavity (Fig. 3left) and LV cavity (Fig. 3right) allowed the surgeon to better understand the precise structure and plan the appropriate repair. In particular, 3-D images of the orifice from outside of LV cavity (Fig. 3left), which clearly visualized the posteromedial papillary muscle through the hole, were much useful to decide the suture site. The TEE image also showed minimal functional mitral regurgitation.

**Fig. 4:**
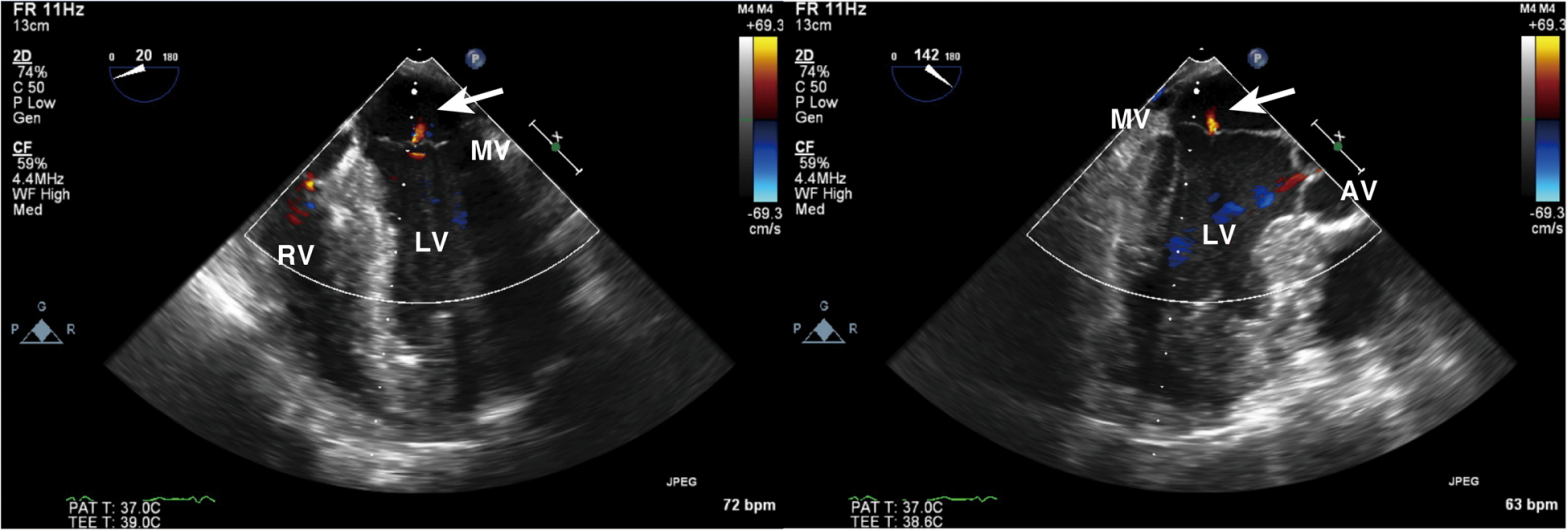
Color-flow Doppler images of mid-esophageal four chamber view (left) and midesophageal long axis view (right) after weaning from cardiopulmonary bypass. There is trivial mitral regurgitation (arrow) after the repair. AV aortic valve, LV left ventricle, MV mitral valve, RV right ventricle

**Fig. 5:**
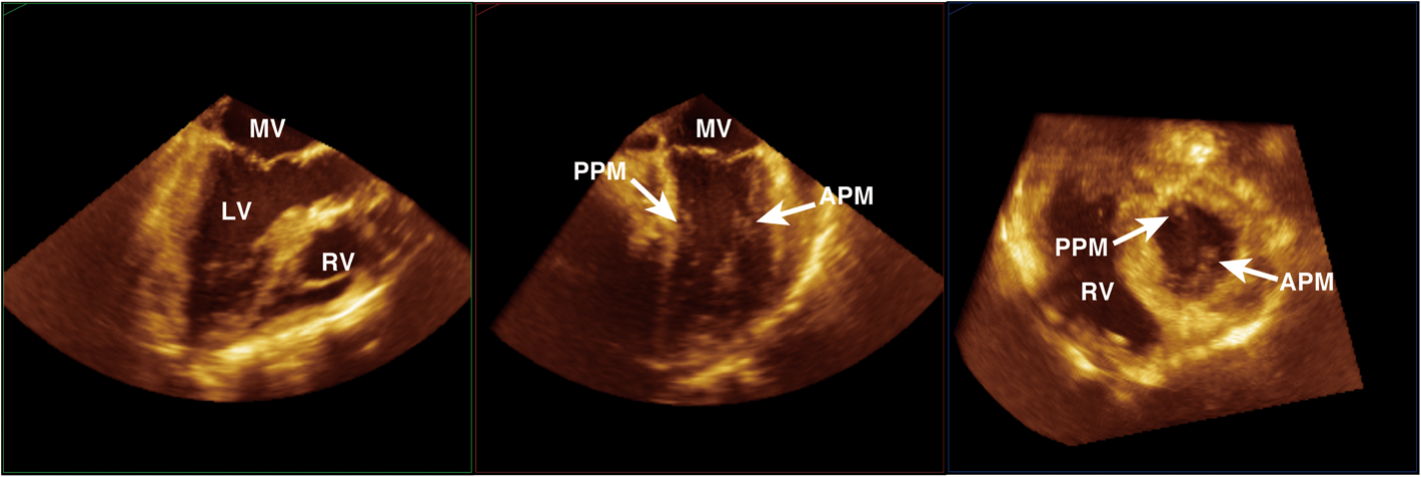
Mid-esophageal long axis view (left), mid-esophageal mitral commissural view (middle), and short axis view of the left ventricle (right). These images were obtained thorugh multiplanar reconstruction from a volume dataset after weaning from cardiopulmonary bypass. The morphology of the mitral valve and posteromedial papillary muscle is normal. APM anterolateral papillary muscle, LV left ventricle, MV mitral valve, PPM posteromedial papillary muscle RV right ventricle

After a cardiopulmonary bypass (CPB) was established through femoral arteriovenous cannulation, a median sternotomy was performed, and adhesions were carefully detached to reach the LV-PAN. There was an extensive necrosis of the epicardium and a hematoma between the epicardium and the muscle layer were confirmed. A 2 × 2-cm defect of the LV wall formed the orifice of the PAN. The defect was closed carefully using the double patch technique to avoid injury of the papillary muscle. The patient was smoothly weaned from cardiopulmonary bypass with dopamine at 3 μg/kg/min, dobutamine at 3 μg/kg/min, milrinone at 0.5 μg/kg/min, and IABP. The surgery was completed, with difficulty in achieving hemostasis. TEE after weaning from CPB revealed neither exacerbation of the mitral regurgitation nor any remarkable changes in the morphology of the mitral valve (Figs. 4 and 5). The postoperative course was favorable and the patient was discharged in an ambulatory state on postoperative day 18.

## Discussion

In the present case, the patient previously underwent aortic root surgery without direct manipulation of the rupture site. There are few reports of cases of LV-PAN formation after aortic root surgery; one reported case described LV-PAN in the LV outflow tract [8]. Injury of the LV by the venting catheter could be a cause of PAN formation; however, the early onset of LV-PAN formation should be expected [9]. Although chronic myocardial ischemia due to the surgical procedure might also be a cause of LV-PAN, no culprit coronary lesion was found and the patient’s LV function after the previous surgery was normal without any regional wall motion abnormality. Thus, an obvious cause of LV-PAN formation could not be determined.

Intraoperative RT 3-D TEE could add spatial information to 2-D TEE and demonstrate the proximity of the rupture site to the posteromedial papillary muscle. Some authors reported that 3-D echocardiography could add spatial information, including the location of the LV rupture, orifice geometry, and complex intracardiac flow, in comparison with 2-D echocardiography [10, 11]. The spatial relationship between the orifice and the papillary muscle is crucial in the surgical repair of LV-PAN, as the repair procedure can induce direct papillary muscle injury and alter the geometry and function of the papillary muscle and mitral valve [12]. The diagnostic modalities for LV-PAN include TTE, TEE, left ventriculography, cardiac computed tomography, and cardiac magnetic resonance imaging [1]. The strengths of RT 3-D TEE compared to the other modalities are better spatial resolution and the ability to easily obtain diverse images useful for the understanding of the anatomical relationship. In addition, 3-D images can provide better sharing of the spatial findings among surgical team members than 2-D imaging because the spatial relationship of structures obtained by 2-D TEE is subject to each observer’s perception.

The efficient application of RT 3-D TEE in cardiac surgery is still evolving, and it has been shown useful for the evaluation of the native valves, prosthetic valves, and the left ventricle [13]. Intraoperative RT 3-D TEE can be indicated in cases where the precise understanding of the anatomical structures is required for clinical decision-making and where information sharing among the surgical team is critical for conducting adequate procedures. The en face view and multiplanar reconstruction that RT 3-D TEE provides may be suitable for satisfying the surgical team’s needs in such cases.

In conclusion, LV-PAN formation after cardiac surgery can present without direct manipulation of the rupture site and major coronary lesion. Intraoperative RT 3-D TEE can facilitate better understanding of the anatomical relationship between the rupture site and the posteromedial papillary muscle and allow for information sharing to avoid complications during surgical repair.

## Data Availability

The data in this case report are available from the corresponding author on reasonable request.

## Abbreviations

2-D: Two-dimensional
3-D: Three-dimensional
CPB: Cardiopulmonary bypass
IABP: Intraaortic balloon pumping
LV: Left ventricle
LV-PAN: Left ventricular pseudoaneurysm
PAN: Pseudoaneurysm
RT 3-D TEE: Real-time three-dimensional transesophageal echocardiography
TEE: Transesophageal echocardiography
TTE: Transthoracic echocardiography
